# Treatment trends and risks of corticosteroid use in adult primary immune thrombocytopenia: a claims database study in Japan

**DOI:** 10.1007/s12185-024-03897-8

**Published:** 2024-12-12

**Authors:** Hirokazu Kashiwagi, Isao Miura, Naohiko Terasawa, Ken-ichi Iwayama, Yuka Furukawa, Makoto Kanenishi

**Affiliations:** 1 Department of Hematology and Oncology, Osaka University Graduate School of Medicine, Postal Address: 2-2 Yamadaoka, Suita, Osaka 565-0871 Japan; 2 Osaka Red Cross Blood Center, Osaka, Japan; 3 Medical Department, Kissei Pharmaceutical Co., Ltd, Tokyo, Japan; 4 RWE Group Clinical Research Department, Ark Medical Solutions Inc, Tokyo, Japan

**Keywords:** Adverse effect, Corticosteroids, Database study, Immune thrombocytopenia, Treatment pattern

## Abstract

Recent trends in the treatment of primary immune thrombocytopenia (ITP) were investigated using a claims database that included data from 16,161 Japanese patients with ITP collected from April 2014 to August 2022. Of the 4144 adult patients analyzed, 1276 received corticosteroids. The mean and median durations of corticosteroid use were 115.31 and 41 days, respectively. The time to withdrawal of corticosteroids was significantly shorter in 2020 to 2021 than in 2015 to 2019. Additionally, the number of prescriptions for thrombopoietin receptor agonists increased from 2015 to 2021 and exceeded that of corticosteroids in 2021. While these results suggest a trend towards reduction in corticosteroid use in real-world settings in Japan, 12.00% of patients received a corticosteroid dose of ≥ 10 mg/day at Week 12. Furthermore, 23.05% of patients continued to receive corticosteroids at Week 24, indicating that some patients were still receiving long-term corticosteroid treatment. The risk of adverse outcomes was significantly associated with corticosteroid use. In conclusion, new treatment options may lead to more sophisticated ITP management with less corticosteroid use, although further research and reconsideration of clinical practice guidelines is needed.

**Supplementary Information:**

The online version contains supplementary material available at 10.1007/s12185-024-03897-8.

## Introduction

Primary immune thrombocytopenia (ITP) is an autoimmune disease, characterized by a low platelet count and an increased risk of bleeding [1]. The first-line treatment of ITP is corticosteroids (CS). The reference guide for ITP in Japan revised in 2019 recommends using prednisolone (PSL) at an initial dose of 0.5–1 mg/kg/day for 2–4 weeks. Subsequently, PSL is tapered to less than 10 mg/kg/day over 8–12 weeks regardless of any increase in platelet count, and the patient’s progress is monitored at a maintenance dose of PSL ≤ 10 mg/day [2]. Thus, long-term treatment of CS at a PSL dose of less than 10 mg/day is allowed under the guideline in order to maintain the platelet count during follow-up. In contrast, the American Society of Hematology (ASH) 2019 guideline recommends avoiding long-term PSL treatment exceeding 6 weeks [3]. Moreover, the International Consensus Report (ICR) recommends that PSL should be administered at 1 mg/kg for 2 weeks (maximum 3 weeks) and should be tapered even when a response is seen, aiming to stop PSL by 6 weeks (maximum 8 weeks), even if the platelet count drops during the tapering period [4].

CS, although effective, should be administered with caution because they are associated with multiple bothersome adverse effects (AEs) compared to other ITP treatments [5]. The main AEs that occur frequently with CS and are likely to be serious are infection, osteoporosis, diabetes, atherosclerosis, gastrointestinal and neuropsychiatric disorders [6]. In addition, 33 CS-related clinical symptoms, which are not life-threatening AEs but can affect health-related quality of life (HRQoL), have also been identified [7]. Although the European League Against Rheumatism (EULAR) reported that the risk of harm in long-term treatment below 5 mg/day was low in most patients [8], even low doses of CS can lead to fracture and infection when used concomitantly with immunosuppressants [9, 10]. EULAR also reported that treatment of PSL between > 5 and ≤ 10 mg/day has the potential to cause harm depending on patient-specific characteristics [8]. Therefore, given that the incidence of infections, diabetes, and vascular events in patients with ITP was higher than in non-ITP subjects [11], patients with ITP may be more susceptible to adverse consequences from CS treatment. Furthermore, both the symptoms and treatments of ITP impact HRQoL [12], with several reports highlighting the impact of CS treatment on HRQoL in patients with ITP [5, 7, 11, 13, 14]. Therefore, the appropriate use of CS should be promoted.

For CS-resistant or dependent patients with ITP, thrombopoietin receptor agonists (TPO-RAs), rituximab (RTX) and splenectomy have been recommended as second-line treatments in recently published guidelines including the Japanese guidelines [2, 3]. In Japan, two TPO-RAs, eltrombopag (EPAG) and romiplostim (ROMI), were approved in 2010 and 2011, respectively, and RTX was approved in 2017. However, real-world data about the transition of ITP treatments after the approval of these drugs in Japan remains very limited [15].

In this study, we investigated recent treatment patterns and the duration and dose of CS treatment in patients with ITP using a Japanese database. This database includes information on many older subjects, for whom morbidity of ITP is high [16]. Moreover, we explored the incidence of AEs associated with CS use in patients with ITP. The aim of this study was to clarify the issues associated with the use of CS to treat ITP in the Japanese context, and to capture the changes in treatment patterns over time. Furthermore, we assessed the influence of new treatment options on CS prescription in real-world practice.

## Methods

### Data source

The administrative claims data were provided by DeSC Healthcare Inc., Tokyo, Japan. This database consists of anonymous information obtained from receipt data and specific health checkup data: the National Health Insurance for self-employed individuals, retired individuals, and their dependents (Kokuho); the association/union-administered health insurance for salaried employees in large companies (Kempo); and the Advanced Elderly Medical Service System for all individuals aged 75 years or older [Koki Koreisya Iryo Seido]. The distribution of gender and age, and the diagnosis of disease in the database have been confirmed to be similar to Japanese national-level statistical data such as the population estimates and National Health and Nutrition Survey [17]. The claims data were collected from more than 11 million subscribers from April 2014 to August 2022.

The study was approved by the Institutional Review Board of MINS, a specified nonprofit corporation and conducted in accordance with the principles of the Declaration of Helsinki. This study protocol was registered in UMIN Clinical Trials Registry (Trial Number: UMIN000052806). The requirement for informed consent was waived because of the anonymity of patient data.

### Study population

**Fig. 1:**
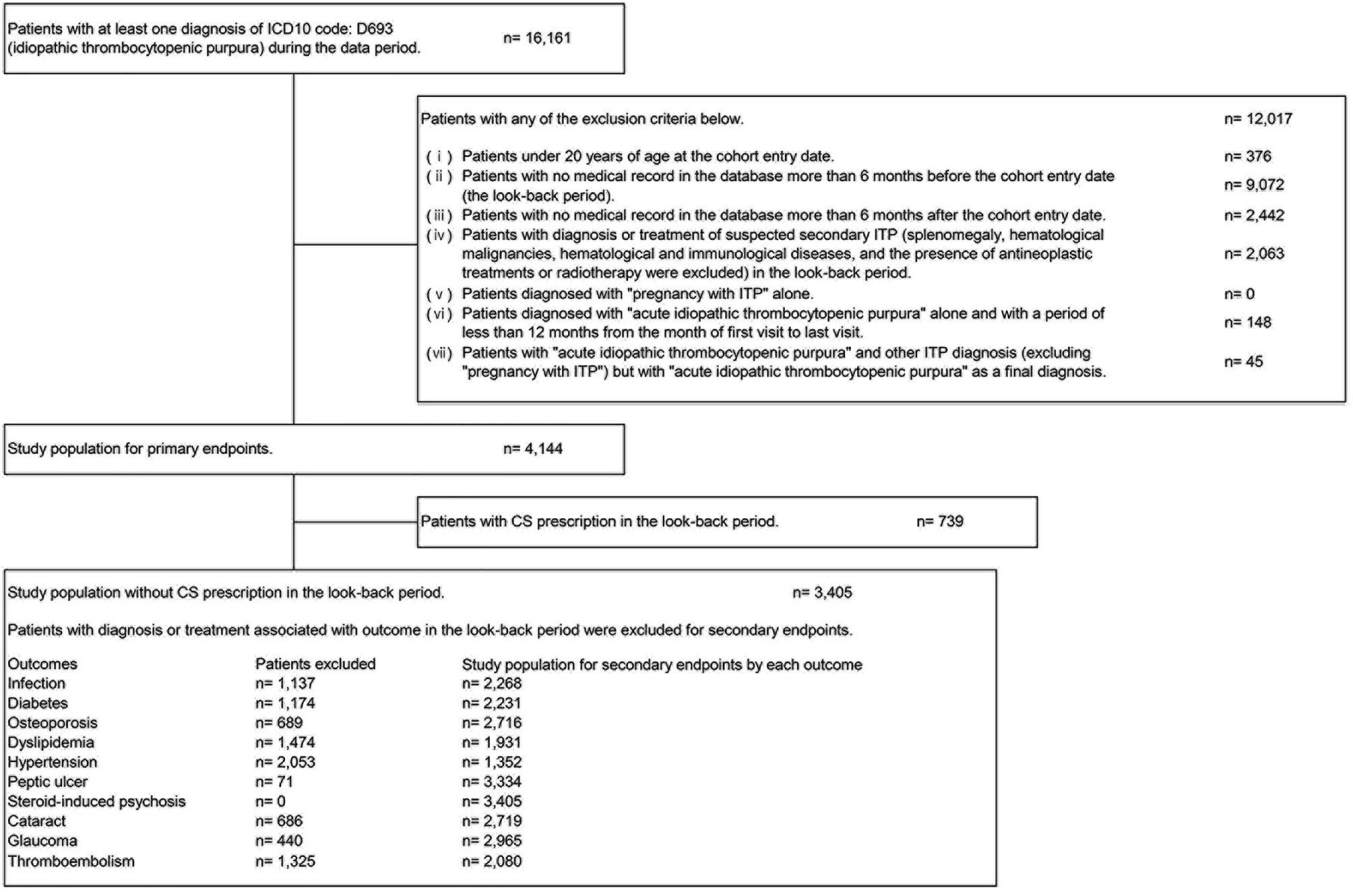
Patient selection flowchart. *CS* corticosteroids, *ICD10* International Statistical Classification of Diseases and Related Health Problems, 10th Revision, *ITP* immune thrombocytopenia

We selected patients who met the inclusion criteria and did not fall under the exclusion criteria as shown in Fig. 1.

For the diagnosis of diseases included in the criteria, suspected disease was not included. The look-back period (6 months before the cohort entry date) is needed to define the baseline population. In this study, for secondary endpoints, the look-back period was set to exclude steroid prevalent users and patients who met the defined exclusion criteria for each disease assessed in the adverse outcome assessment shown in Supplementary Table 1.

### Primary outcome

Target drugs and treatments are presented in Supplementary Table 2. The primary outcome was recent trends in ITP treatment (CS prescriptions, change in treatment over time, and treatment patterns). The following data were collected: baseline patient characteristics at the cohort entry date, comorbidities in the look-back period, and prescription status of CS and other treatments during the evaluation period. In the assessment of prescribed CS dose, except for methylprednisolone pulse therapy (prescription was from 0.5 to 1.0 g/day for 3 consecutive days as injection alone not oral) and high-dose dexamethasone therapy (prescription was from 20 to 40 mg/day for 4 consecutive days as oral or injection), information about “corticosteroid” prescribed within the same ID as the provisional receipt ID with the ITP disease code was collected. The dose of CS was calculated as the equivalent value of PSL. The assessment period was from the initial diagnosis of ITP to the last day the disease name code was recorded. The visit month with the disease name code of “pregnancy with ITP” was not included in the assessment period.

### Secondary outcome

The secondary outcome was the risk of adverse outcomes associated with CS prescription. A nested case–control study was conducted. The target diseases were as follows: infection, diabetes, osteoporosis with compression fracture and/or femur head necrosis (hereinafter called osteoporosis), dyslipidemia, hypertension, peptic ulcer with gastrointestinal bleeding (hereinafter called peptic ulcer), steroid-induced psychosis, cataract, glaucoma and thromboembolism (myocardial infarction, angina pectoris, stroke, cerebral hemorrhage, peripheral arterial disease, venous thromboembolism).

The cases, which were matched with at least one control, were patients with target outcomes after the first diagnosis of ITP. Controls were selected using risk-set sampling, which is the least biased method for control sampling in a nested case–control study. That is, controls were selected from the non-case patients during the at-risk period at the occurrence of each case by time-point matching. It was permissible for a future case to be selected as a control more than once for different cases. The matching ratio of controls was limited to a maximum of five per case, and controls were randomly sampled when the ratio exceeded five. After matching, background factors in cases and controls were summarized for each outcome. As covariates, information on gender and age was collected at the cohort entry date, and information on disease-related covariates was collected in the look-back period. The assessment period was from the date of first diagnosis for ITP (cohort entry date) to the earlier date in the first occurrence of the outcomes or the end of observation.

### Definitions

The gap period of each drug for treatment pattern analysis is shown in Supplementary Table 3. In consecutive prescriptions of the same treatments, when the interval from the end date of the last prescription to the next prescription date was less than or equal to the gap period, the prescription periods were combined and regarded as a single continuous prescription period. For injectable drugs, when the interval between consecutive prescription dates was within the gap period, the continuous prescription periods was defined as above.

The definition of outcomes is presented in Supplementary Table 4. In every case, the outcome was determined when these definitions were applied for the first time.

### Data analysis

Summary statistics were provided for duration of CS prescription. The mean dose of CS (mg/day) in patients who received a CS prescription was calculated in each week from the initiation of prescription (the initial prescription, every 2 weeks until Week 20, and then Weeks 24, 28, 52, 104, and 156). In this analysis, the number of patients with a CS dose of 0 mg/day was defined as the number of patients with no CS prescription.

The number and proportion of patients who received a CS, TPO-RAs, RTX or underwent splenectomy were calculated by year (2015–2021). Prescription and splenectomy multiple times within each year was counted as one time (one case).

In patients who discontinued CS, the time to withdrawal of CS in the 6 months after initiation of CS was evaluated using the Kaplan–Meier method with the log-rank test applied for comparisons by year. In this analysis, CS treatment was determined to be withdrawn if days from the end date of CS prescription [prescription date + number of prescription days] to the next CS prescription date was 60 days or more, or if CS were not prescribed for no fewer than 60 days from the date of the last CS prescription to the evaluation end date.

The prescription proportion for each treatment line was determined by the treatment sequence (Supplementary Fig. 1). On the other hand, based on the period of each prescription, the treatment pattern was determined by the switching and adding on of treatments (Supplementary Fig. 2).

Logistic regression analysis was performed to evaluate the association between the duration of CS prescription or cumulative dose of CS and the adverse outcome. Multivariate logistic regression was used to control for potential confounders of gender, age, malignancy, pulmonary disease, and liver disease. The odds ratio and 95% confidence interval were calculated.

### Statistics

For categorical data, frequencies and proportions were described. Summary statistics (number, mean, standard deviation, median, first quartile, third quartile, minimum value, maximum value, median time to withdrawal of CS) were calculated for numerical data. The significance level was set at 5% (two-sided). Confidence intervals were two-sided, and the confidence level was 95%. Missing values were not supplemented with estimated or calculated values. All statistical analyses were performed using SAS Enterprise Guide and System Release 9.4 (SAS® Institute, Inc., Cary, NC, USA).

## Results

### Study population and characteristics

Of 16,161 subjects with at least one diagnosis of ITP, 4,144 patients were included in this study (Fig. 1). At baseline, the median (interquartile range) age was 78 (68–84) years, 48.99% (2,030/4,144) were male, and 23.77% (985/4,144) were inpatients. The most common comorbidity was hypertension (58.23% [2,413/4,144]), followed by dyslipidemia (43.12% [1,787/4,144]) and diabetes (35.57% [1,474/4,144]). Overall, 50.27% (2,083/4,144) had received at least one treatment for ITP (Table 1). Among patients with treatment for ITP, 61.26% (1,276/2,083) of patients received CS prescription (Table 2).

**Table 1:** Baseline characteristics

	All patients	Treatment for ITP	
		Yes	No
All patients, n (%)	4144 (100.00)	2083 (50.27)	2061 (49.73)
Gender, n (%)			
Female	2114 (51.01)	1034 (49.64)	1080 (52.40)
Male	2030 (48.99)	1049 (50.36)	981 (47.60)
Age, years			
Mean	74.61	76.53	72.68
Standard deviation	13.86	12.47	14.90
Median	78	79	77
IQR			
25 percentile	68	70	67
75 percentile	84	85	83
Min	20	20	20
Max	101	98	101
Inpatient/outpatient, n (%)			
Inpatient	985 (23.77)	856 (41.09)	129 (6.26)
Outpatient	3159 (76.23)	1227 (58.91)	1932 (93.74)
Comorbidities^a^, n (%)			
Diabetes	1474 (35.57)	721 (34.61)	753 (36.54)
Malignancy	936 (22.59)	485 (23.28)	451 (21.88)
Pulmonary disease	1116 (26.93)	621 (29.81)	495 (24.02)
Liver disease	926 (22.35)	428 (20.55)	498 (24.16)
Osteoporosis^b^	945 (22.80)	501 (24.05)	444 (21.54)
Dyslipidemia	1787 (43.12)	866 (41.57)	921 (44.69)
Hypertension	2413 (58.23)	1255 (60.25)	1158 (56.19)
Cataract	882 (21.28)	471 (22.61)	411 (19.94)
Glaucoma	548 (13.22)	291 (13.97)	257 (12.47)
Infection	927 (22.37)	481 (23.09)	446 (21.64)
Myocardial infarction	75 (1.81)	35 (1.68)	40 (1.94)
Angina pectoris	752 (18.15)	373 (17.91)	379 (18.39)
Stroke	491 (11.85)	272 (13.06)	219 (10.63)
Cerebral hemorrhage	69 (1.67)	41 (1.97)	28 (1.36)
Peripheral arterial disease	117 (2.82)	64 (3.07)	53 (2.57)
Venous thromboembolism	121 (2.92)	51 (2.45)	70 (3.40)
Peptic ulcer^c^	96 (2.32)	54 (2.59)	42 (2.04)
Depression	295 (7.12)	151 (7.25)	144 (6.99)
Insomnia	1,126 (27.17)	573 (27.51)	553 (26.83)

^a^Duplicate count

^b^Osteoporosis refers to osteoporosis with compression fracture and/or femur head necrosis

^c^Peptic ulcer refers to peptic ulcer with gastrointestinal bleeding

*CS* corticosteroids, *IQR* interquartile range, *ITP* immune thrombocytopenia

**Table 2:** Summary of CS prescription

	Descriptive statistics
All patients, n (%)	4144 (100.00)
Duration of CS prescription (days), n (%)	
Untreated	2868 (69.21)
> 0– < 60	785 (18.94)
≥ 60– < 90	128 (3.09)
≥ 90– < 180	149 (3.60)
≥ 180	214 (5.16)
Duration of CS prescription (days)	
Mean	115.31
Standard deviation	211.37
Median	41
IQR	
25 percentile	18
75 percentile	107
Min	1
Max	2219

*CS* corticosteroids, *IQR* interquartile range

### Duration of CS prescription

The mean and median (interquartile range) duration of CS prescription were 115.31 and 41 (18–107) days, respectively. Among patients with ITP, 30.79% (1,276/4,144) received CS prescription, and among patients who received CS prescription, 28.45% (363/1,276) received CS for more than 90 days (Table 2).

### Dose of CS prescription

Among patients who received CS prescription, 42.24% (539/1,276), 19.51% (249/1,276), and 10.74% (137/1,276) were prescribed ≥ 20– < 40 mg/day, ≥ 40– < 60 mg/day, and ≥ 60 mg/day as the initial CS dose, respectively. At Week 2 and Week 4, 21.68% (274/1,264) and 44.33% (551/1,243), respectively, were not prescribed CS. On the other hand, 34.08% of patients received CS at Week 8 and the prescription continuation rates by dose at Week 12 was 9.36% (110/1,175) for ≥ 10– < 20 mg/day, 2.47% (29/1,175) for ≥ 20– < 40 mg/day, 0% (0/1,175) for ≥ 40– < 60 mg/day, and 0.17% (2/1,175) for ≥ 60 mg/day. Furthermore, 23.05% (251/1,089) of patients had not discontinued CS at Week 24 (Table 3).

**Table 3:** Prescribed CS dose at each time point

Time point (Week)	Initial	2	4	6	8	10	12	14	16	18	20	24	28	52	104	156
All^a^, n	1276	1264	1243	1220	1203	1189	1175	1161	1147	1136	1121	1089	1055	822	479	238
CS dose (mg/day), n (%)																
0	– (–)	274 (21.68)	551 (44.33)	696 (57.05)	793 (65.92)	831 (69.89)	837 (71.23)	847 (72.95)	852 (74.28)	866 (76.23)	843 (75.20)	838 (76.95)	739 (70.05)	601 (73.11)	393 (82.05)	192 (80.67)
> 0– < 2.5	56 (4.39)	57 (4.51)	54 (4.34)	49 (4.02)	37 (3.08)	45 (3.78)	41 (3.49)	42 (3.62)	42 (3.66)	44 (3.87)	57 (5.08)	55 (5.05)	161 (15.26)	153 (18.61)	58 (12.11)	33 (13.87)
≥ 2.5– < 5.0	66 (5.17)	68 (5.38)	56 (4.51)	56 (4.59)	55 (4.57)	37 (3.11)	46 (3.91)	51 (4.39)	59 (5.14)	55 (4.84)	74 (6.60)	66 (6.06)	76 (7.20)	34 (4.14)	13 (2.71)	9 (3.78)
≥ 5.0– < 7.5	56 (4.39)	67 (5.30)	66 (5.31)	64 (5.25)	55 (4.57)	65 (5.47)	72 (6.13)	80 (6.89)	75 (6.54)	68 (5.99)	54 (4.82)	58 (5.33)	46 (4.36)	27 (3.28)	7 (1.46)	0 (0.00)
≥ 7.5– < 10	31 (2.43)	50 (3.96)	41 (3.30)	43 (3.52)	41 (3.41)	48 (4.04)	38 (3.23)	36 (3.10)	33 (2.88)	33 (2.90)	32 (2.85)	32 (2.94)	12 (1.14)	3 (0.36)	2 (0.42)	3 (1.26)
≥ 10– < 20	142 (11.13)	230 (18.20)	193 (15.53)	162 (13.28)	146 (12.14)	118 (9.92)	110 (9.36)	88 (7.58)	73 (6.36)	60 (5.28)	55 (4.91)	36 (3.31)	21 (1.99)	4 (0.49)	6 (1.25)	0 (0.00)
≥ 20– < 40	539 (42.24)	371 (29.35)	231 (18.58)	124 (10.16)	70 (5.82)	42 (3.53)	29 (2.47)	13 (1.12)	10 (0.87)	7 (0.62)	4 (0.36)	2 (0.18)	0 (0.00)	0 (0.00)	0 (0.00)	1 (0.42)
≥ 40– < 60	249 (19.51)	112 (8.86)	41 (3.30)	22 (1.80)	6 (0.50)	2 (0.17)	0 (0.00)	2 (0.17)	2 (0.17)	2 (0.18)	2 (0.18)	1 (0.09)	0 (0.00)	0 (0.00)	0 (0.00)	0 (0.00)
≥ 60	137 (10.74)	35 (2.77)	10 (0.80)	4 (0.33)	0 (0.00)	1 (0.08)	2 (0.17)	2 (0.17)	1 (0.09)	1 (0.09)	0 (0.00)	1 (0.09)	0 (0.00)	0 (0.00)	0 (0.00)	0 (0.00)

^a^Patients prescribed CS at least once were counted

*CS* corticosteroids

### Time to withdrawal of CS

**Fig. 2:**
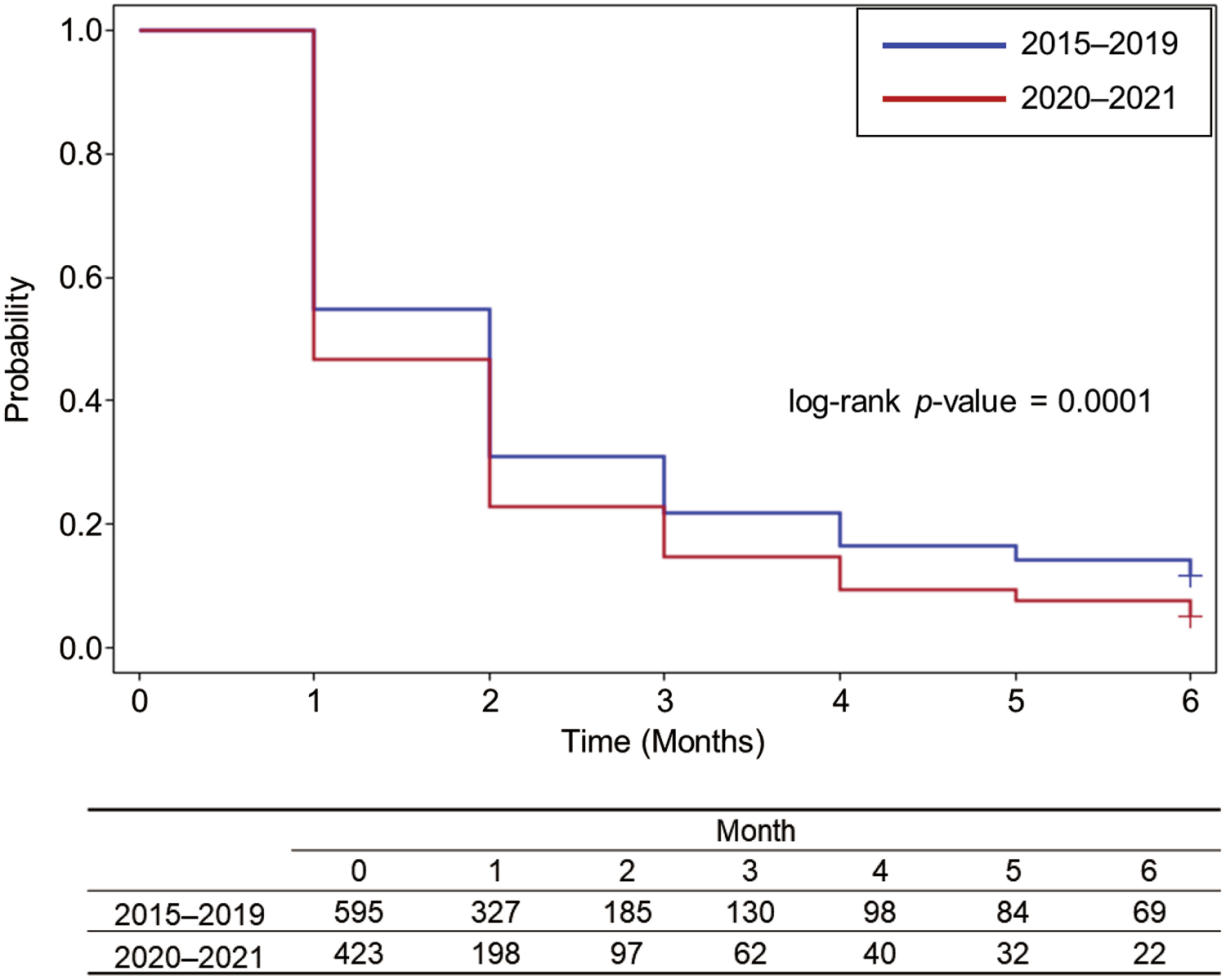
Kaplan–Meier curve for the assessment of the time until withdrawal of CS within 6 months. CS, corticosteroids

Median time to withdrawal of CS (interquartile range) was 35 (18–79) days in 2015–2019 and 28 (15–57) days in 2020–2021. Median time to withdrawal of CS (95% CI) was 35 (32–38) and 28 (24–32) days, respectively. In 2015–2019, of 595 patients, 54.96% (327/595) and 21.85% (130/595) received CS prescription at 1 month and 3 months, respectively, after the start of CS treatment. On the other hand, in 2020–2021, of 423 patients, 46.81% (198/423) and 14.66% (62/423) received CS prescription at 1 month and 3 months, respectively, after the start of CS treatment. The time to withdrawal of CS in 2020–2021 was significantly shorter than that in 2015–2019 (*p* = 0.0001) (Fig. 2).

### Changes in ITP treatment over time

**Fig. 3:**
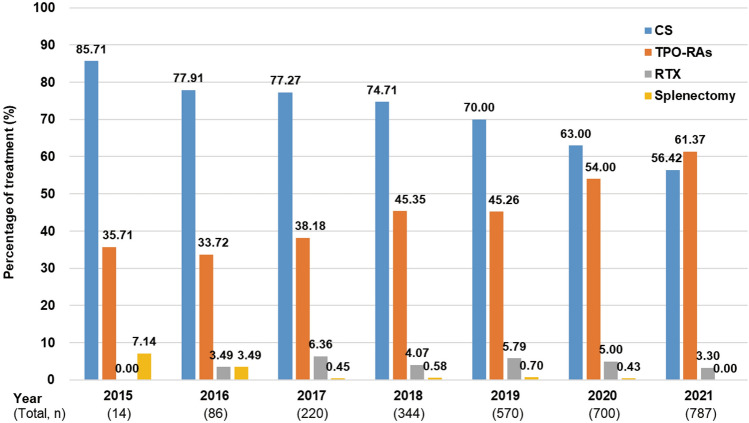
Changes in ITP treatment over time. *CS* corticosteroids, *ITP* immune thrombocytopenia, *RTX* rituximab, *TPO-RAs* thrombopoietin receptor agonists

The proportions of patients who received CS prescription were 77.91% (67/86), 70.00% (399/570), and 56.42% (444/787) in 2016, 2019 and 2021, respectively. For TPO-RAs, the proportions were 33.72% (29/86), 45.26% (258/570), and 61.37% (483/787) in 2016, 2019 and 2021, respectively. The proportion of patients receiving RTX remained at 3 to 6% after 2016. The proportion of patients undergoing splenectomy was less than 1% after 2017 (Fig. 3).

### Treatment patterns in patients with ITP

Table 4 shows the prescription rate by treatment line. To ascertain the treatment preference, the sequence of treatments was investigated. As first-line treatment, 73.49% (1,128/1,535) of patients were prescribed CS alone, and for TPO-RAs alone, the figure was 17.52% (269/1,535: EPAG 246, ROMI 23). In a small number of patients, the combination of CS, TPO-RAs and other immunosuppressants was observed as first-line treatment. As second-line treatment, 69.95% (405/579: EPAG 375, ROMI 30) of patients were prescribed TPO-RAs. RTX appeared to be mostly used as third-line treatment in 29.41% (40/136) of patients. Among these treatment lines, few patients underwent splenectomy.

**Table 4:** Sequence of treatment line

	Sequence of treatment		
	1st	2nd	3rd
All, n (%)	1535 (100.00)	579 (100.00)	136 (100.00)
Treatment, n (%)			
CS	1128 (73.49)	89 (15.37)	14 (10.29)
CS|EPAG	24 (1.56)	0 (0.00)	0 (0.00)
CS|EPAG|Others^a^	1 (0.07)	0 (0.00)	0 (0.00)
CS|ROMI	3 (0.20)	0 (0.00)	0 (0.00)
CS|ROMI|Others^a^	1 (0.07)	0 (0.00)	0 (0.00)
CS|RTX	5 (0.33)	1 (0.17)	0 (0.00)
CS|Splenectomy	0 (0.00)	1 (0.17)	0 (0.00)
CS|Others^a^	6 (0.39)	2 (0.35)	1 (0.74)
EPAG	246 (16.03)	375 (64.77)	20 (14.71)
EPAG|RTX	0 (0.00)	0 (0.00)	1 (0.74)
EPAG|Others^a^	9 (0.59)	2 (0.35)	0 (0.00)
ROMI	23 (1.50)	30 (5.18)	26 (19.12)
ROMI|Others^a^	1 (0.07)	1 (0.17)	0 (0.00)
RTX	13 (0.85)	38 (6.56)	40 (29.41)
Splenectomy	2 (0.13)	2 (0.35)	7 (5.15)
Others^a^	73 (4.76)	38 (6.56)	27 (19.85)

^a^Others include azathioprine, cyclophosphamide, cyclosporine, diaphenylsulfone, danazol, mycophenolate mofetil, vinca alkaloid and their combination

*CS* corticosteroids, *EPAG* eltrombopag, *ROMI* romiplostim, *RTX* rituximab

**Fig. 4:**
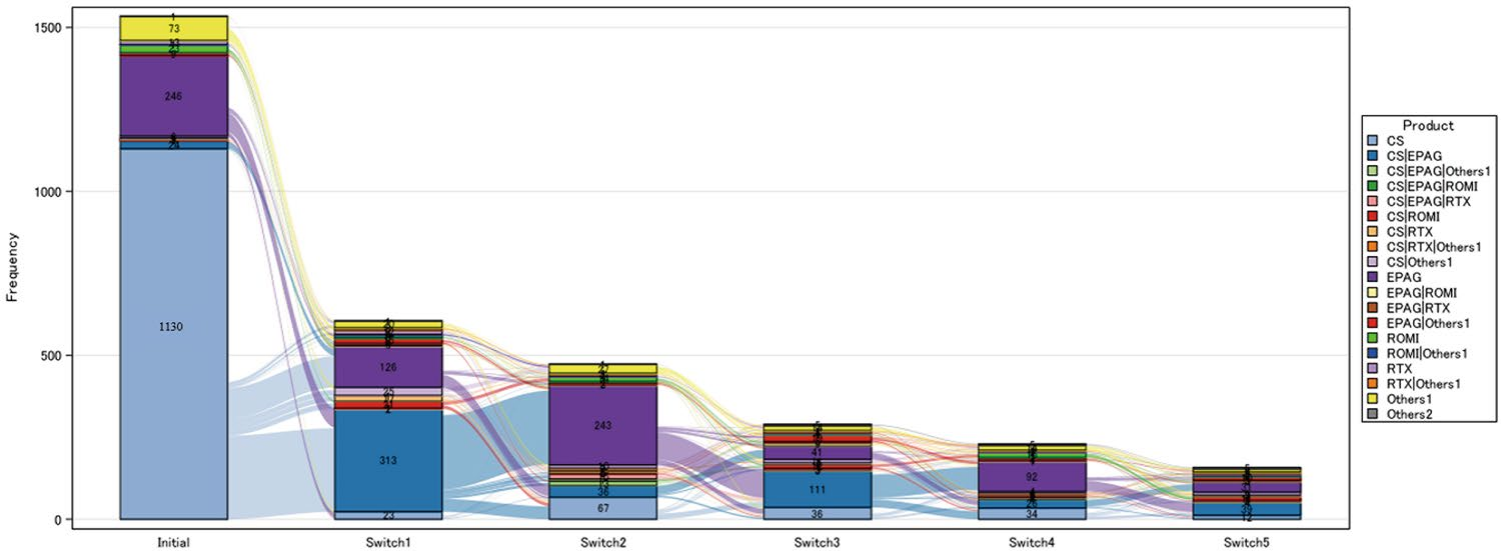
Treatment switching patterns. The details on patient numbers of each bar are presented in Supplementary Table 5. Others1 include azathioprine, cyclophosphamide, cyclosporine, diaphenylsulfone, danazol, mycophenolate mofetil, vinca alkaloid, and their combination. Treatment patterns in which the total number of cases from the initial to 5th switch is less than 5 (CS|EPAG|ROMI|Others1, CS|EPAG|RTX|Others1, CS|EPAG|ROMI|RTX, CS|ROMI |Others1, CS|ROMI|RTX, EPAG|RTX|Others1, ROMI|RTX) are included in Others2. CS, corticosteroids; EPAG, eltrombopag; ROMI, romiplostim; RTX, rituximab

Furthermore, the Sankey diagram shows the switching of prescriptions in patients with ITP (Fig. 4). To ascertain the details of switch and add-on of prescriptions in each patient, every change in prescription pattern was investigated as a treatment switch. Among the patients who were prescribed CS alone as the initial treatment (n = 1,130), 419 patients switched prescription in the first switching. In these patients, 60.86% (255/419) of patients added EPAG, and 22.67% (95/419) switched to EPAG alone. In the second switching, 71.88% (225/313) of patients who were treated with CS plus EPAG (CS|EPAG) switched to EPAG alone. However, CS were re-administrated in about one third of patients who received EPAG alone in the second switching (33.74% [82/243]), and CS were shown to be administered repeatedly in some cases (Fig. 4, Supplementary Table 5).

### Association between CS prescription and the risk of adverse outcomes

The incidence of each adverse outcome was as follows: 25.57% (580/2,268) for infections, 5.02% (112/2,231) for diabetes, 4.31% (117/2,716) for osteoporosis, 3.68% (71/1,931) for dyslipidemia, 13.09% (177/1,352) for hypertension, 6.63% (221/3,334) for peptic ulcer, 7.69% (209/2,719) for cataract, 1.59% (47/2,965) for glaucoma, and 7.36% (153/2,080) for thromboembolism. Because only a few instances of steroid-induced psychosis occurred, it was excluded from the analysis (Supplementary Table 6).

The association between the risk of any adverse outcome and the presence of CS prescription was statistically significant (glaucoma; *p* = 0.0160, other than glaucoma; *p* < 0.0001 in the multivariate model) (Table 5). There was no association between outcome risk and the duration of CS prescription or cumulative dose of CS except for peptic ulcer (Tables 6, 7).

**Table 5:** Association with the risk of adverse outcomes and the presence of CS prescription, logistic regression analysis

Outcomes		Cases, n	Controls, n	Univariate model		Multivariate model^a^	
				Odds ratio [95% CI]	*p* value	Odds ratio [95% CI]	*p* value
Infection	Number of patients	580	2,900				
	CS prescription						
	No	290	2,165	Reference		Reference	
	Yes	290	735	2.97 [2.47, 3.58]	< 0.0001	2.90 [2.40, 3.50]	< 0.0001
Diabetes	Number of patients	112	560				
	CS prescription						
	No	21	368	Reference		Reference	
	Yes	91	192	9.83 [5.70, 16.95]	< 0.0001	9.74 [5.63, 16.88]	< 0.0001
Osteoporosis^b^	Number of patients	117	585				
	CS prescription						
	No	38	355	Reference		Reference	
	Yes	79	230	3.64 [2.31, 5.75]	< 0.0001	3.18 [1.95, 5.18]	< 0.0001
Dyslipidemia	Number of patients	71	355				
	CS prescription						
	No	23	209	Reference		Reference	
	Yes	48	146	3.23 [1.83, 5.70]	< 0.0001	3.25 [1.80, 5.88]	< 0.0001
Hypertension	Number of patients	177	885				
	CS prescription						
	No	80	567	Reference		Reference	
	Yes	97	318	2.33 [1.65, 3.29]	< 0.0001	2.21 [1.55, 3.17]	< 0.0001
Peptic ulcer^c^	Number of patients	221	1,105				
	CS prescription						
	No	86	709	Reference		Reference	
	Yes	135	396	3.25 [2.35, 4.50]	< 0.0001	3.18 [2.29, 4.43]	< 0.0001
Cataract	Number of patients	209	1,045				
	CS prescription						
	No	77	574	Reference		Reference	
	Yes	132	471	2.10 [1.54, 2.87]	< 0.0001	2.05 [1.50, 2.81]	< 0.0001
Glaucoma	Number of patients	47	235				
	CS prescription						
	No	19	141	Reference		Reference	
	Yes	28	94	2.39 [1.21, 4.73]	0.0122	2.39 [1.18, 4.84]	0.0160
Thromboembolism	Number of patients	153	765				
	CS prescription						
	No	70	495	Reference		Reference	
	Yes	83	270	2.30 [1.59, 3.32]	< 0.0001	2.16 [1.48, 3.14]	< 0.0001

^a^Adjusted for gender, age, comorbidities; malignancy, pulmonary disease, liver disease

^b^Osteoporosis refers to osteoporosis with compression fracture and/or femur head necrosis

^c^Peptic ulcer refers to peptic ulcer with gastrointestinal bleeding

CI, confidence interval; CS, corticosteroids

**Table 6:** Association with the risk of adverse outcomes and duration of CS prescription, logistic regression analysis

Outcomes		Cases, n	Controls, n	Univariate model		Multivariate model^a^	
				Odds ratio [95% CI]	*p* value	Odds ratio [95% CI]	*p* value
Infection	Number of patients	580	2,900				
	Duration of CS (days)						
	Untreated	290	2,165	Reference		Reference	
	> 0– < 15	141	329	3.21 [2.54, 4.07]	< 0.0001	3.25 [2.55, 4.14]	< 0.0001
	≥ 15– < 60	72	132	4.28 [3.08, 5.95]	< 0.0001	4.05 [2.89, 5.68]	< 0.0001
	≥ 60	77	274	2.09 [1.57, 2.78]	< 0.0001	1.96 [1.46, 2.62]	< 0.0001
Diabetes	Number of patients	112	560				
	Duration of CS (days)						
	Untreated	21	368	Reference		Reference	
	> 0– < 15	42	73	9.93 [5.51, 17.90]	< 0.0001	9.85 [5.45, 17.80]	< 0.0001
	≥ 15– < 60	18	39	9.33 [4.27, 20.37]	< 0.0001	9.33 [4.22, 20.61]	< 0.0001
	≥ 60	31	80	10.03 [4.89, 20.56]	< 0.0001	9.87 [4.74, 20.52]	< 0.0001
Osteoporosis^b^	Number of patients	117	585				
	Duration of CS (days)						
	Untreated	38	355	Reference		Reference	
	> 0– < 15	17	70	2.55 [1.34, 4.85]	0.0045	2.18 [1.09, 4.34]	0.0266
	≥ 15– < 60	12	30	4.18 [1.92, 9.11]	0.0003	4.63 [1.95, 10.99]	0.0005
	≥ 60	50	130	4.18 [2.51, 6.95]	< 0.0001	3.51 [2.03, 6.09]	< 0.0001
Dyslipidemia	Number of patients	71	355				
	Duration of CS (days)						
	Untreated	23	209	Reference		Reference	
	> 0– < 15	13	45	2.66 [1.23, 5.76]	0.0133	2.86 [1.29, 6.37]	0.0100
	≥ 15– < 60	10	28	3.47 [1.45, 8.29]	0.0051	3.46 [1.42, 8.43]	0.0063
	≥ 60	25	73	3.59 [1.79, 7.21]	0.0003	3.49 [1.67, 7.28]	0.0009
Hypertension	Number of patients	177	885				
	Duration of CS (days)						
	Untreated	80	567	Reference		Reference	
	> 0– < 15	42	116	2.74 [1.76, 4.27]	< 0.0001	2.52 [1.60, 3.97]	< 0.0001
	≥ 15– < 60	21	62	2.56 [1.43, 4.58]	0.0016	2.86 [1.55, 5.26]	0.0008
	≥ 60	34	140	1.84 [1.14, 2.94]	0.0118	1.64 [1.00, 2.69]	0.0479
Peptic ulcer^c^	Number of patients	221	1,105				
	Duration of CS (days)						
	Untreated	86	709	Reference		Reference	
	> 0– < 15	71	147	4.23 [2.90, 6.17]	< 0.0001	4.23 [2.88, 6.22]	< 0.0001
	≥ 15– < 60	29	75	3.63 [2.14, 6.15]	< 0.0001	3.47 [2.04, 5.92]	< 0.0001
	≥ 60	35	174	1.84 [1.15, 2.95]	0.0111	1.74 [1.08, 2.82]	0.0234
Cataract	Number of patients	209	1,045				
	Duration of CS (days)						
	Untreated	77	574	Reference		Reference	
	> 0– < 15	36	139	1.95 [1.26, 3.01]	0.0027	1.98 [1.27, 3.09]	0.0025
	≥ 15– < 60	15	76	1.47 [0.81, 2.68]	0.2083	1.41 [0.77, 2.58]	0.2669
	≥ 60	81	256	2.39 [1.68, 3.38]	< 0.0001	2.29 [1.60, 3.27]	< 0.0001
Glaucoma	Number of patients	47	235				
	Duration of CS (days)						
	Untreated	19	141	Reference		Reference	
	> 0– < 15	9	32	2.22 [0.90, 5.50]	0.0843	2.22 [0.88, 5.57]	0.0905
	≥ 15– < 60	6	18	2.60 [0.92, 7.33]	0.0700	2.54 [0.86, 7.47]	0.0902
	≥ 60	13	44	2.43 [1.05, 5.61]	0.0378	2.47 [1.03, 5.93]	0.0433
Thromboembolism	Number of patients	153	765				
	Duration of CS (days)						
	Untreated	70	495	Reference		Reference	
	> 0– < 15	33	104	2.34 [1.45, 3.78]	0.0005	2.25 [1.39, 3.66]	0.0011
	≥ 15– < 60	22	53	3.05 [1.71, 5.43]	0.0002	2.71 [1.50, 4.87]	0.0009
	≥ 60	28	113	1.83 [1.10, 3.07]	0.0211	1.72 [1.02, 2.91]	0.0434

^a^Adjusted for gender, age, comorbidities; malignancy, pulmonary disease, liver disease

^b^Osteoporosis refers to osteoporosis with compression fracture and/or femur head necrosis

^c^Peptic ulcer refers to peptic ulcer with gastrointestinal bleeding

CI, confidence interval; CS, corticosteroids

**Table 7:** Association with the risk of adverse outcomes and cumulative dose of CS, logistic regression analysis

Outcomes				Univariate model		Multivariate model^a^	
		Cases, n	Controls, n	Odds ratio [95% CI]	*p* value	Odds ratio [95% CI]	*p* value
Infection	Number of patients	580	2,900				
	Cumulative dose of CS (mg)						
	Untreated	290	2,165	Reference		Reference	
	> 0– < 500	133	324	3.08 [2.43, 3.90]	< 0.0001	3.08 [2.42, 3.92]	< 0.0001
	≥ 500– < 1500	66	173	2.88 [2.11, 3.93]	< 0.0001	2.64 [1.92, 3.62]	< 0.0001
	≥ 1500	91	238	2.90 [2.20, 3.82]	< 0.0001	2.85 [2.15, 3.79]	< 0.0001
Diabetes	Number of patients	112	560				
	Cumulative dose of CS (mg)						
	Untreated	21	368	Reference		Reference	
	> 0– < 500	35	69	9.24 [4.97, 17.20]	< 0.0001	9.15 [4.89, 17.09]	< 0.0001
	≥ 500– < 1500	24	40	11.59 [5.62, 23.87]	< 0.0001	11.15 [5.34, 23.25]	< 0.0001
	≥ 1500	32	83	9.28 [4.67, 18.42]	< 0.0001	9.55 [4.75, 19.21]	< 0.0001
Osteoporosis^b^	Number of patients	117	585				
	Cumulative dose of CS (mg)						
	Untreated	38	355	Reference		Reference	
	> 0– < 500	20	60	3.65 [1.94, 6.87]	< 0.0001	2.97 [1.51, 5.85]	0.0016
	≥ 500– < 1500	12	48	2.50 [1.21, 5.18]	0.0138	1.87 [0.86, 4.08]	0.1167
	≥ 1500	47	122	4.19 [2.51, 6.98]	< 0.0001	4.13 [2.35, 7.27]	< 0.0001
Dyslipidemia	Number of patients	71	355				
	Cumulative dose of CS (mg)						
	Untreated	23	209	Reference		Reference	
	> 0– < 500	8	42	1.81 [0.74, 4.42]	0.1930	1.95 [0.78, 4.90]	0.1529
	≥ 500– < 1500	13	33	3.77 [1.68, 8.46]	0.0013	3.68 [1.60, 8.45]	0.0022
	≥ 1500	27	71	3.92 [2.01, 7.64]	< 0.0001	3.90 [1.95, 7.79]	0.0001
Hypertension	Number of patients	177	885				
	Cumulative dose of CS (mg)						
	Untreated	80	567	Reference		Reference	
	> 0– < 500	35	111	2.40 [1.52, 3.79]	0.0002	2.28 [1.42, 3.65]	0.0006
	≥ 500– < 1500	14	75	1.38 [0.73, 2.60]	0.3176	1.32 [0.69, 2.52]	0.4002
	≥ 1500	48	132	2.90 [1.88, 4.48]	< 0.0001	2.78 [1.77, 4.36]	< 0.0001
Peptic ulcer^c^	Number of patients	221	1,105				
	Cumulative dose of CS (mg)						
	Untreated	86	709	Reference		Reference	
	> 0– < 500	69	137	4.43 [3.03, 6.49]	< 0.0001	4.44 [3.02, 6.54]	< 0.0001
	≥ 500– < 1500	24	85	2.54 [1.48, 4.33]	0.0007	2.43 [1.41, 4.17]	0.0013
	≥ 1500	42	174	2.31 [1.48, 3.61]	0.0002	2.21 [1.40, 3.48]	0.0007
Cataract	Number of patients	209	1,045				
	Cumulative dose of CS (mg)						
	Untreated	77	574	Reference		Reference	
	> 0– < 500	38	136	2.13 [1.38, 3.28]	0.0006	2.17 [1.40, 3.39]	0.0006
	≥ 500– < 1500	16	91	1.30 [0.73, 2.33]	0.3770	1.25 [0.69, 2.26]	0.4552
	≥ 1500	78	244	2.41 [1.70, 3.43]	< 0.0001	2.31 [1.61, 3.30]	< 0.0001
Glaucoma	Number of patients	47	235				
	Cumulative dose of CS (mg)						
	Untreated	19	141	Reference		Reference	
	> 0– < 500	11	35	2.51 [1.07, 5.90]	0.0353	2.51 [1.05, 6.02]	0.0387
	≥ 500– < 1500	3	16	1.53 [0.41, 5.70]	0.5300	1.61 [0.42, 6.20]	0.4885
	≥ 1500	14	43	2.66 [1.17, 6.02]	0.0192	2.56 [1.09, 6.04]	0.0313
Thromboembolism	Number of patients	153	765				
	Cumulative dose of CS (mg)						
	Untreated	70	495	Reference		Reference	
	> 0– < 500	32	98	2.45 [1.50, 4.01]	0.0003	2.32 [1.41, 3.82]	0.0009
	≥ 500– < 1500	13	53	1.82 [0.94, 3.54]	0.0766	1.73 [0.87, 3.44]	0.1169
	≥ 1500	38	119	2.40 [1.51, 3.81]	0.0002	2.21 [1.38, 3.55]	0.0010

^a^Adjusted for gender, age, comorbidities; malignancy, pulmonary disease, liver disease

^b^Osteoporosis refers to osteoporosis with compression fracture and/or femur head necrosis

^c^Peptic ulcer refers to peptic ulcer with gastrointestinal bleeding

CI, confidence interval; CS, corticosteroids

## Discussion

Using a Japanese claims database, the present study revealed the current status of treatment for ITP in real-world settings including recent trends in treatment patterns, detailed information on CS usage, and the association between the risk of AEs and CS prescription.

The database used in this study primarily included subjects aged over 65 years, and the median age of subjects in this database was 78 (Table 1). These results indicate that this study revealed the actual status of CS prescription with the focus on older populations in whom ITP is more likely to occur. Mean duration of CS prescription was 115.31 days, indicating that some patients were still receiving long-term treatment with CS. Meanwhile, the median duration of CS prescription was 41 days, comparable to the ASH 2019 guideline (the ASH 2019 guideline recommends against a prolonged course exceeding 6 weeks including treatment and taper) (Table 2) [3]. The proportion of patients not prescribed CS at Week 2 and Week 4 was 21.68% and 44.33%, respectively (Table 3). However, among patients who discontinued CS by Week 4, the proportion of patients who did not switch drugs or were not re-administered CS was 25% (314/1,264) (data not shown), comparable to the previously reported discontinuation rate of CS [2].

The prescription rate of CS decreased over time from 2015 to 2021 (Fig. 3). This may be mostly due to the prevalence of new agents such as TPO-RAs, which were described as a second-line therapy in the 2019 revision of the Japanese guideline [2], and partly due to the increased awareness of the AEs associated with CS through the release of the ASH 2019 guideline [3]. In fact, the time to withdrawal of CS in 2020–2021 was significantly shorter than that in 2015–2019 (Fig. 2). In addition, the prescription of TPO-RAs increased over time from 2015 to 2021 particularly after 2019 and exceeded CS prescriptions in 2021 (Fig. 3). These results indicate a trend towards a reduction in CS use in real-world settings in Japan. In contrast, our results also showed that 34.08% received CS at Week 8 (ICR guideline recommends a maximum of 8 weeks) and 12.00% received a dose of ≥ 10 mg/day at Week 12 [4], suggesting that a considerable proportion of patients with ITP need not a low dose of CS for prolonged period. Furthermore, 23.05% (251/1089) did not discontinue CS at Week 24 (Table 3). These results suggest that a certain number of patients continued to receive long-term treatment with CS due to the lack of other treatment options. Further treatment options need to become available to allow successful tapering in these patients because the risk of harm is significant in long-term CS treatment [18].

Although CS were primarily prescribed as first-line treatment, TPO-RAs were also prescribed from the beginning in a substantially high proportion of patients as previously reported [15], suggesting that the AEs caused by CS are probably being taken into consideration, especially when CS is administered to older people. Among TPO-RAs, EPAG is used more often than ROMI probably due to the convenience of using EPAG. The majority of patients who changed treatments from CS alone as the initial treatment added EPAG as the first switch. Then, in the second switch, most of patients who were receiving CS plus EPAG (CS|EPAG) switched to EPAG alone. Interestingly, as the third switch, CS were re-administered to about one third of patients who had previously received EPAG alone. In addition, CS were shown to be administered repeatedly after discontinuation (Fig. 4). These results indicate that CS are used as a rescue therapy or that some patients are dependent on CS. The patients who are unable to use or increase the dose of TPO-RAs may be dependent on CS. The reasons why TPO-RAs are difficult to use or use at higher doses include the risk of thrombosis, which is a notable AE associated with TPO-RAs, fluctuations in platelet count, and barriers to medication adherence [2]. It should be noted that patients with ITP are likely to be at high risk for thrombosis including being positive for antiphospholipid antibodies [19–22], and treatment with TPO-RAs may further accentuate toward greater thrombogenicity by increasing the risk factors for thrombosis [23].

The present study confirmed that the risk of any adverse outcomes assessed in this study was significantly increased by CS prescription (Table 5). However, no increased risk of CS-related AEs proportional to treatment duration or cumulative dose was shown. Some outcomes showed that the lowest risk was in the mid-term range, such as a treatment duration of ≥ 15– < 60 days and a cumulative dose of ≥ 500– < 1,500 mg (Tables 6, 7). Price et al. reported that short-term CS treatment can cause nearly the same types of AEs as long-term CS treatment, and that even one short-course CS has the potential to increase the risk of AEs [24]. The cumulative dose of CS treatment also influences the risk of AEs [25]. Therefore, in the treatment of ITP with CS followed by tapering, the risk of CS-related AEs might have been high in the early phase with a high dose and in the late phase with a high cumulative dose. In one cohort study, it was reported that in patients with ITP, mortality caused by infections was more common than by bleeding [26], suggesting that CS should be administered with caution taking into consideration the impact of AEs on prognosis. Moreover, the impact of CS on HRQoL should also be taken into account in patients with ITP, considering the fact that the incidence of ITP in females is much higher than that males in young population and females experience higher levels of bother than males on CS treatment [5, 16]. Taken together, it is necessary to further raise awareness regarding the appropriate use of CS, and earlier transition to second-line treatment is recommended.

This study had some limitations due to the reliance on receipt data for analysis: The definition of each disease registered in the database might differ from the actual clinical diagnosis and condition of patients. We were unable to obtain the information about the reasons for prescribing particular agents. Therefore, the treatment of interest could be administered for reasons other than ITP, especially in case of CS. Though there are other types of insurers in Japan, the databases used in this study only covered three types of insurers and included a large proportion of older subjects aged over 65 years [17]. In addition, in this study, a 6-month look-back period was set to define new steroid users and patients with no history of the target disease. As a result, more than 9,000 patients were excluded because of a lack of medical records in the look-back period (Fig. 1). While this prevented the inclusion of non-newly diagnosed patients due to insurance switching, a certain number of young people and patients with few comorbidities may have been excluded. These factors might cause selection bias. Since data from each type of insurance were not linked, some patients might have been duplicated in more than one set of insurance data. It should be noted that more than 25% patients were started on low-dose CS of less than 20 mg/day (Table 3), which is not described in the guidelines. This may reflect the real-world practice of Japanese doctors, but it may also be a result of this limitation. That is, although this study attempted to analyze patients newly diagnosed with ITP, patients who stopped treatment once outside the follow-up period and whose condition worsened again after an interval of more than 6 months may be included as newly diagnosed patients since it was not possible to confirm treatment outside the database period (no data before 2014) or before the insurance switching (insurance data is not linked). In such cases low-dose CS might be used if a good response to CS was expected according to the previous medical records. The number of eligible patients in the earlier years of the study periods was smaller than in subsequent years. This might affect the prescription rate by year. It was not possible to discuss the influence of patient characteristics not included in the data such as alcohol intake and smoking status.

In conclusion, this study revealed recent trends in the treatment of ITP. Although a certain number of patients still received long-term treatment with CS, there was a trend towards the reduction and early discontinuation of CS use in real-world settings in Japan. The findings of this study suggest that new treatment options, such as TPO-RAs, may be the underlying reason for this trend. However, some patients with ITP experience difficulty in using TPO-RAs because of the high risk of thrombosis, which is a notable AE associated with TPO-RAs. Furthermore, in addition to the trend towards long-term CS treatment as mentioned above, this study confirmed that CS use was an important risk factor for many AEs. Additional research and raising awareness of the potential for adverse outcomes with CS in ITP treatment are necessary to further optimize treatment strategies and improve patient outcomes. Recently, many new molecular targeted agents such as spleen tyrosine kinase inhibitors and neonatal Fc receptor inhibitors have been developed as new treatment options for ITP, and furthermore other agents of new mechanism of action such as Bruton’s tyrosine kinase inhibitors and CD38 monoclonal antibody are under development. In this context, the Spanish Working Group for ITP has updated its main recommendations including the clinical approach for patients at high risk of thromboembolism [27]. It is expected that these new alternative treatment options can contribute to the development of more individualized therapy based on each patient’s medical history and clinical status, leading to more sophisticated ITP management with less CS use.

## Supplementary Information

Below is the link to the electronic supplementary material.

Supplementary file1 (DOCX 97 KB)

## Data Availability

The data analyzed in this study is subject to the following licenses/restrictions: the data that support the findings of this study are available from DeSC Healthcare, Inc. (Tokyo, Japan) but restrictions apply to the availability of these data, which were used under license for the current study, and so are not publicly available. Data are however available from the authors upon reasonable request and with permission of DeSC Healthcare, Inc. Requests to access these datasets should be directed to Kissei Pharmaceutical Co., Ltd.
