# Correction: Treatment trends and risks of corticosteroid use in adult primary immune thrombocytopenia: a claims database study in Japan

**DOI:** 10.1007/s12185-025-03931-3

**Published:** 2025-01-31

**Authors:** Hirokazu Kashiwagi, Isao Miura, Naohiko Terasawa, Ken-ichi Iwayama, Yuka Furukawa, Makoto Kanenishi

**Affiliations:** 1 Department of Hematology and Oncology, Osaka University Graduate School of Medicine, Postal Address: 2-2 Yamadaoka, Suita, Osaka 565-0871 Japan; 2 Osaka Red Cross Blood Center, Osaka, Japan; 3 Medical Department, Kissei Pharmaceutical Co., Ltd, Tokyo, Japan; 4 RWE Group Clinical Research Department, Ark Medical Solutions Inc, Tokyo, Japan

**Correction: International Journal of Hematology** 10.1007/s12185-024-03897-8

The article ‘Treatment trends and risks of corticosteroid use in adult primary immune thrombocytopenia: a claims database study in Japan’, written by Hirokazu Kashiwagi, Isao Miura, Naohiko Terasawa, Ken‑ichi Iwayama, Yuka Furukawa, Makoto Kanenishi, was originally published under exclusive license to Japanese Society of Hematology. As a result of the subsequent decision to publish the article under the open access model, the article’s copyright notice was changed on 30 December 2024 to © The Author(s) 2025 and the article is now distributed under a Creative Commons Attribution 4.0 International License, which permits use, sharing, adaptation, distribution and reproduction in any medium or format, as long as you give appropriate credit to the original author(s) and the source, provide a link to the Creative Commons licence, and indicate if changes were made. The images or other third party material in this article are included in the article’s Creative Commons licence, unless indicated otherwise in a credit line to the material. If material is not included in the article’s Creative Commons licence and your intended use is not permitted by statutory regulation or exceeds the permitted use, you will need to obtain permission directly from the copyright holder. To view a copy of this licence, visit http://creativecommons.org/licenses/by/4.0/.

The original article has been corrected.

