# Supplementary material for: Treatment trends and risks of corticosteroid use in adult primary immune thrombocytopenia: a claims database study in Japan
Source: Int J Hematol. 2024 Dec 12;121(3):363–77. doi: 10.1007/s12185-024-03897-8 (PMC11861122; doi:10.1007/s12185-024-03897-8)
Supplement: Supplementary file 1 — Supplementary file1 (DOCX 97 KB) [file 12185_2024_3897_MOESM1_ESM.docx]

**Supplementary information**

Treatment trends and risks of corticosteroid use in adult primary immune thrombocytopenia: A claims database study in Japan

Hirokazu Kashiwagi^1,2^, Isao Miura^3^, Naohiko Terasawa^3^, Ken-ichi Iwayama^3^, Yuka Furukawa^4^, Makoto Kanenishi^4^

^1^Department of Hematology and Oncology, Osaka University Graduate School of Medicine, Osaka, Japan

^2^Osaka Red Cross Blood Center, Osaka, Japan

^3^Medical Department, Kissei Pharmaceutical Co., Ltd., Tokyo, Japan

^4^RWE Group Clinical Research Department, Ark Medical Solutions Inc., Tokyo, Japan

Corresponding Author: Hirokazu Kashiwagi

Supplementary Table 1. Secondary outcomes and the exclusion criteria established for case-control study

^a^Suspicious cases in the diagnosis were excluded.

^b^Osteoporosis refers to osteoporosis with compression fracture and/or femur head necrosis.

| **Outcomes^a^** | **Exclusion criteria** |
| --- | --- |
| Infection | Patients prescribed antibacterial, antiviral, antifungal, or tuberculosis agents at cohort entry and immunosuppressive agents in the look-back period. |
| Diabetes | Patients diagnosed with diabetes or prescribed treatments in the look-back period. |
| Osteoporosis^b^ | Patients diagnosed with osteoporosis or compression fracture/femur head necrosis in the look-back period. |
| Dyslipidemia | Patients diagnosed with dyslipidemia or prescribed treatments in the look-back period. |
| Hypertension | Patients diagnosed with hypertension or prescribed treatments in the look-back period. |
| Peptic ulcer^c^ | Patients diagnosed with peptic ulcer with gastrointestinal bleeding in the look-back period. |
| Steroid-induced psychosis | Patients diagnosed with steroid-induced psychosis in the look-back period. |
| Cataract | Patients diagnosed with cataract, prescribed treatments or underwent surgery in the look-back period. |
| Glaucoma | Patients diagnosed with glaucoma, prescribed treatments or underwent surgery in the look-back period. |
| Thromboembolism | Patients diagnosed thromboembolism or prescribed treatments in the look-back period. |

^c^Peptic ulcer refers to peptic ulcer with gastrointestinal bleeding.

Supplementary Table 2. Target drugs and treatment

| **Category** | **Drugs, Treatments** |
| --- | --- |
| CS | [Primary outcomes]  dexamethasone, methylprednisolone, prednisolone  [Secondary outcomes]  ATC code: all corticosteroids for systemic use to meet H02AB and H02BX |
|  |  |
| TPO-RAs | romiplostim, eltrombopag |
| Anti-CD20 monoclonal antibody | rituximab |
| Other immunosuppressants | azathioprine, cyclosporine, cyclophosphamide, diaphenylsulfone,  danazol, vinca alkaloid, mycophenolate mofetil |
| Other | splenectomy |

ATC, anatomical therapeutic chemical classification system; CS, corticosteroids; TPO-RAs, thrombopoietin receptor agonists.

Supplementary Table 3. Gap period of each drug

| **Drugs** | **Gap period** |
| --- | --- |
| steroid (oral/injection) | 7 days |
| romiplostim | 21 days |
| eltrombopag | 14 days |
| rituximab | 21 days ※up to 4 consecutive times |
| azathioprine | 7 days |
| cyclosporine (oral/injection) | 7 days |
| cyclophosphamide (oral) | 7 days |
| cyclophosphamide (injection) | 28 days |
| diaphenylsulfone | 7 days |
| danazol | 7 days |
| vinca alkaloid | 28 days |
| mycophenolate mofetil | 7 days |

Supplementary Table 4. Definitions of outcomes

^a^Osteoporosis refers to osteoporosis with compression fracture and/or femur head necrosis.

^b^Peptic ulcer refers to peptic ulcer with gastrointestinal bleeding.

| **Outcomes** | **Definition** |
| --- | --- |
| Infection | disease + drugs |
| Diabetes | disease + drugs |
| Osteoporosis^a^ | disease (osteoporosis + compression fracture and/or femur head necrosis) |
| Dyslipidemia | disease + drugs |
| Hypertension | disease + drugs |
| Peptic ulcer^b^ | disease + drugs |
| Steroid-induced psychosis | disease |
| Cataract | disease + drugs/operation |
| Glaucoma | disease + drugs/operation |
| Thromboembolism | disease + drugs |

Supplementary Table 5. Treatment switching patterns.

|  | **Initial** | **Switch1** | **Switch2** | **Switch3** | **Switch4** | **Switch5** |
| --- | --- | --- | --- | --- | --- | --- |
| **All, N (%)** | 1,535 (100.00) | 606 (100.00) | 474 (100.00) | 290 (100.00) | 230 (100.00) | 158 (100.00) |
| **Treatment, n (%)** |  |  |  |  |  |  |
| CS | 1,130 (73.62) | 23 (3.80) | 67 (14.14) | 36 (12.41) | 34 (14.78) | 12 (7.59) |
| CS\|EPAG | 24 (1.56) | 313 (51.65) | 36 (7.59) | 111 (38.28) | 26 (11.30) | 39 (24.68) |
| CS\|EPAG\|Others1 | 1 (0.07) | 1 (0.17) | 13 (2.74) | 3 (1.03) | 4 (1.74) | 4 (2.53) |
| CS\|EPAG\|ROMI | 0 (0.00) | 0 (0.00) | 7 (1.48) | 0 (0.00) | 2 (0.87) | 0 (0.00) |
| CS\|EPAG\|ROMI\|Others1^a^ | 0 (0.00) | 0 (0.00) | 0 (0.00) | 1 (0.34) | 0 (0.00) | 1 (0.63) |
| CS\|EPAG\|RTX | 0 (0.00) | 2 (0.33) | 15 (3.16) | 5 (1.72) | 5 (2.17) | 2 (1.27) |
| CS\|EPAG\|RTX\|Others1^a^ | 0 (0.00) | 0 (0.00) | 0 (0.00) | 1 (0.34) | 0 (0.00) | 1 (0.63) |
| CS\|EPAG\|ROMI\|RTX^a^ | 0 (0.00) | 0 (0.00) | 0 (0.00) | 2 (0.69) | 0 (0.00) | 0 (0.00) |
| CS\|ROMI | 3 (0.20) | 21 (3.47) | 5 (1.05) | 12 (4.14) | 3 (1.30) | 11 (6.96) |
| CS\|ROM I\|Others1^a^ | 1 (0.07) | 1 (0.17) | 0 (0.00) | 0 (0.00) | 1 (0.43) | 1 (0.63) |
| CS\|ROMI\|RTX^a^ | 0 (0.00) | 0 (0.00) | 1 (0.21) | 1 (0.34) | 2 (0.87) | 0 (0.00) |
| CS\|RTX | 5 (0.33) | 17 (2.81) | 6 (1.27) | 4 (1.38) | 1 (0.43) | 3 (1.90) |
| CS\|RTX\|Others1^a^ | 0 (0.00) | 1 (0.17) | 7 (1.48) | 1 (0.34) | 6 (2.61) | 3 (1.90) |
| CS\|Others1 | 6 (0.39) | 25 (4.13) | 10 (2.11) | 11 (3.79) | 4 (1.74) | 9 (5.70) |
| EPAG | 246 (16.03) | 126 (20.79) | 243 (51.27) | 41 (14.14) | 92 (40.00) | 31 (19.62) |
| EPAG\|ROMI | 0 (0.00) | 5 (0.83) | 2 (0.42) | 9 (3.10) | 1 (0.43) | 3 (1.90) |
| EPAG\|RTX | 0 (0.00) | 4 (0.66) | 3 (0.63) | 5 (1.72) | 4 (1.74) | 3 (1.90) |
| EPAG\|RTX\|Others1 | 0 (0.00) | 0 (0.00) | 0 (0.00) | 0 (0.00) | 2 (0.87) | 0 (0.00) |
| EPAG\|Others1 | 9 (0.59) | 15 (2.48) | 7 (1.48) | 19 (6.55) | 7 (3.04) | 10 (6.33) |
| ROMI | 23 (1.50) | 8 (1.32) | 14 (2.95) | 4 (1.38) | 15 (6.52) | 4 (2.53) |
| ROMI\|RTX^a^ | 0 (0.00) | 0 (0.00) | 0 (0.00) | 0 (0.00) | 0 (0.00) | 2 (1.27) |
| ROMI\|Others1 | 1 (0.07) | 4 (0.66) | 1 (0.21) | 1 (0.34) | 1 (0.43) | 2 (1.27) |
| RTX | 13 (0.85) | 12 (1.98) | 7(1.48) | 1 (0.34) | 2 (0.87) | 3 (1.90) |
| RTX\|Others1 | 0 (0.00) | 8 (1.32) | 3 (0.63) | 8 (2.76) | 4 (1.74) | 5 (3.16) |
| Others1 | 73 (4.67) | 20 (3.30) | 27 (5.70) | 14 (4.83) | 14 (6.09) | 9 (5.70) |

CS, corticosteroids; EPAG, eltrombopag; ROMI, romiplostim; RTX, rituximab.

Others1 includes azathioprine, cyclophosphamide, cyclosporine, diaphenylsulfone, danazol, mycophenolate mofetil, vinca alkaloid, and their combination.

^a^These are included in Others2 in Figure 4.

Supplementary Table 6. Characteristics of cases and controls

|  | **Infection** | | **Diabetes** | | **Osteoporosis^c^** | | | **Dyslipidemia** | | **Hypertension** | |
| --- | --- | --- | --- | --- | --- | --- | --- | --- | --- | --- | --- |
|  | **Cases** | **Controls** | **Cases** | **Controls** | **Cases** | | **Controls** | **Cases** | **Controls** | **Cases** | **Controls** |
| **All, N** | 580 | 2,900 | 112 | 560 | 117 | | 585 | 71 | 355 | 177 | 885 |
| **Gender, n (%)** |  |  |  |  |  | |  |  |  |  |  |
| Male | 303 (52.24) | 1391 (47.97) | 52 (46.43) | 255 (45.54) | 45 (38.46) | | 320 (54.70) | 40 (56.34) | 161 (45.35) | 93 (52.54) | 398 (44.97) |
| **Age at cohort entry (years)** | | | | | |  |  |  |  |  |  |
| Mean | 77.71 | 72.25 | 75.93 | 72.52 | 79.98 | | 71.34 | 70.65 | 71.51 | 73.55 | 66.21 |
| Standard deviation | 11.92 | 14.00 | 11.41 | 15.74 | 8.36 | | 14.42 | 11.30 | 15.59 | 11.45 | 16.56 |
| Median | 80 | 76 | 79 | 78 | 80 | | 75 | 69 | 77 | 76 | 69 |
| IQR |  |  |  |  |  | |  |  |  |  |  |
| 25 percentile | 72 | 66 | 69 | 66 | 76 | | 66 | 64 | 65 | 66 | 58 |
| 75 percentile | 86 | 82 | 84 | 84 | 85 | | 82 | 80 | 82 | 83 | 79 |
| Min | 21 | 20 | 31 | 21 | 47 | | 20 | 39 | 21 | 39 | 20 |
| Max | 98 | 100 | 93 | 99 | 98 | | 96 | 91 | 97 | 98 | 99 |
| **Duration from cohort entry until index date (months)**^a^ | | | | | |  |  |  |  |  |  |
| Mean | 13.30 | 13.30 | 8.64 | 8.64 | 16.75 | | 16.75 | 14.15 | 14.15 | 13.74 | 13.74 |
| Standard deviation | 13.39 | 13.39 | 12.88 | 12.83 | 15.88 | | 15.83 | 14.38 | 14.30 | 16.25 | 16.22 |
| Median | 8 | 8 | 3 | 3 | 12 | | 12 | 12 | 12 | 7 | 7 |
| IQR |  |  |  |  |  | |  |  |  |  |  |
| 25 percentile | 2 | 2 | 0 | 0 | 4 | | 4 | 1 | 1 | 1 | 1 |
| 75 percentile | 20 | 20 | 11.5 | 11.5 | 25 | | 25 | 23 | 23 | 22 | 22 |
| Min | 1 | 1 | 0 | 0 | 0 | | 0 | 0 | 0 | 0 | 0 |
| Max | 72 | 72 | 65 | 65 | 66 | | 66 | 57 | 57 | 80 | 80 |
| **Comorbidities at cohort entry, n (%)^b^** | | | | | | | | | | | |
| Diabetes | 200 (34.48) | 947 (32.66) | 0 (0.00) | 0 (0.00) | 39 (33.33) | | 177 (30.26) | 17 (23.94) | 71 (20.00) | 31 (17.51) | 156 (17.63) |
| Malignancy | 139 (23.97) | 573 (19.76) | 20 (17.86) | 106 (18.93) | 26 (22.22) | | 110 (18.80) | 7 (9.86) | 73 (20.56) | 20 (11.30) | 148 (16.72) |
| Pulmonary disease | 154 (26.55) | 602 (20.76) | 24 (21.43) | 139 (24.82) | 34 (29.06) | | 120 (20.51) | 13 (18.31) | 74 (20.85) | 30 (16.95) | 149 (16.84) |
| Liver disease | 129 (22.24) | 612 (21.10) | 20 (17.86) | 95 (16.96) | 21 (17.95) | | 117 (20.00) | 2 (2.82) | 50 (14.08) | 26 (14.69) | 134 (15.14) |
| Osteoporosis^c^ | 124 (21.38) | 499 (17.21) | 21 (18.75) | 107 (19.11) | 0 (0.00) | | 0 (0.00) | 6 (8.45) | 56 (15.77) | 24 (13.56) | 89 (10.06) |
| Dyslipidemia | 249 (42.93) | 1177 (40.59) | 41 (36.61) | 173 (30.89) | 49 (41.88) | | 222 (37.95) | 0 (0.00) | 0 (0.00) | 27 (15.25) | 176 (19.89) |
| Hypertension | 353 (60.86) | 1579 (54.45) | 57 (50.89) | 266 (47.50) | 67 (57.26) | | 298 (50.94) | 21 (29.58) | 150 (42.25) | 0 (0.00) | 0 (0.00) |
| Cataract | 119 (20.52) | 544 (18.76) | 13 (11.61) | 112 (20.00) | 21 (17.95) | | 109 (18.63) | 11 (15.49) | 71 (20.00) | 25 (14.12) | 116 (13.11) |
| Glaucoma | 72 (12.41) | 361 (12.45) | 12 (10.71) | 61 (10.89) | 14 (11.97) | | 58 (9.91) | 6 (8.45) | 41 (11.55) | 13 (7.34) | 91 (10.28) |
| Infection | 109 (18.79) | 465 (16.03) | 16 (14.29) | 94 (16.79) | 26 (22.22) | | 89 (15.21) | 7 (9.86) | 58 (16.34) | 22 (12.43) | 149 (16.84) |
| Myocardial infarction | 10 (1.72) | 54 (1.86) | 1 (0.89) | 10 (1.79) | 3 (2.56) | | 10 (1.71) | 0 (0.00) | 1 (0.28) | 0 (0.00) | 2 (0.23) |
| Angina pectoris | 113 (19.48) | 465 (16.03) | 17 (15.18) | 63 (11.25) | 26 (22.22) | | 75 (12.82) | 5 (7.04) | 32 (9.01) | 5 (2.82) | 31 (3.50) |
| Stroke | 74 (12.76) | 266 (9.17) | 12 (10.71) | 57 (10.18) | 15 (12.82) | | 65 (11.11) | 4 (5.63) | 22 (6.20) | 9 (5.08) | 30 (3.39) |
| Cerebral hemorrhage | 13 (2.24) | 33 (1.14) | 3 (2.68) | 9 (1.61) | 3 (2.56) | | 9 (1.54) | 2 (2.82) | 3 (0.85) | 1 (0.56) | 0 (0.00) |
| Peripheral arterial disease | 15 (2.59) | 43 (1.48) | 1 (0.89) | 12 (2.14) | 4 (3.42) | | 15 (2.56) | 3 (4.23) | 4 (1.13) | 3 (1.69) | 8 (0.90) |
| Venous thromboembolism | 14 (2.41) | 46 (1.59) | 1 (0.89) | 6 (1.07) | 3 (2.56) | | 6 (1.03) | 0 (0.00) | 5 (1.41) | 2 (1.13) | 11 (1.24) |
| Peptic ulcer^d^ | 17 (2.93) | 34 (1.17) | 0 (0.00) | 13 (2.32) | 2 (1.71) | | 7 (1.20) | 0 (0.00) | 1 (0.28) | 1 (0.56) | 10 (1.13) |
| Depression | 37 (6.38) | 169 (5.83) | 8 (7.14) | 38 (6.79) | 6 (5.13) | | 34 (5.81) | 3 (4.23) | 18 (5.07) | 9 (5.08) | 32 (3.62) |
| Insomnia | 153 (26.38) | 622 (21.45) | 23 (20.54) | 126 (22.50) | 33 (28.21) | | 109 (18.63) | 8 (11.27) | 81 (22.82) | 27 (15.25) | 113 (12.77) |

Supplementary Table 6. Characteristics of cases and controls (Continued)

|  | **Peptic ulcer^d^** | | **Cataract** | | **Glaucoma** | | **Thromboembolism** | |
| --- | --- | --- | --- | --- | --- | --- | --- | --- |
|  | **Cases** | **Controls** | **Cases** | **Controls** | **Cases** | **Controls** | **Cases** | **Controls** |
| **All, N** | 221 | 1,105 | 209 | 1,045 | 47 | 235 | 153 | 765 |
| **Gender, n (%)** |  |  |  |  |  |  |  |  |
| Male | 132 (59.73) | 526 (47.60) | 111 (53.11) | 502 (48.04) | 24 (51.06) | 111 (47.23) | 78 (50.98) | 318 (41.57) |
| **Age at cohort entry (years)** | | | | | | | | |
| Mean | 77.42 | 73.99 | 76.89 | 71.47 | 74.74 | 72.69 | 75.77 | 69.56 |
| Standard deviation | 11.44 | 14.21 | 7.76 | 15.91 | 10.44 | 14.47 | 11.96 | 15.45 |
| Median | 79 | 78 | 78 | 76 | 78 | 76 | 78 | 71 |
| IQR |  |  |  |  |  |  |  |  |
| 25 percentile | 72 | 68 | 71 | 65 | 68 | 66 | 69 | 63 |
| 75 percentile | 85 | 84 | 82 | 83 | 82 | 83 | 85 | 81 |
| Min | 23 | 20 | 50 | 20 | 41 | 21 | 31 | 21 |
| Max | 99 | 100 | 95 | 100 | 95 | 100 | 98 | 100 |
| **Duration from cohort entry until index date (months)**^a^ | | | | | | | | |
| Mean | 11.81 | 11.81 | 17.78 | 17.78 | 16.45 | 16.45 | 14.16 | 14.16 |
| Standard deviation | 14.48 | 14.46 | 14.73 | 14.70 | 15.37 | 15.24 | 14.45 | 14.41 |
| Median | 6 | 6 | 13 | 13 | 12 | 12 | 9 | 9 |
| IQR |  |  |  |  |  |  |  |  |
| 25 percentile | 0 | 0 | 7 | 7 | 2 | 2 | 2 | 2 |
| 75 percentile | 19 | 19 | 24 | 24 | 29 | 29 | 22 | 22 |
| Min | 0 | 0 | 0 | 0 | 0 | 0 | 0 | 0 |
| Max | 70 | 70 | 75 | 75 | 49 | 49 | 75 | 75 |
| **Comorbidities at cohort entry, n (%)^b^** | | | | | | | | |
| Diabetes | 78 (35.29) | 370 (33.48) | 83 (39.71) | 299 (28.61) | 17 (36.17) | 84 (35.74) | 39 (25.49) | 188 (24.58) |
| Malignancy | 50 (22.62) | 222 (20.09) | 39 (18.66) | 188 (17.99) | 7 (14.89) | 50 (21.28) | 33 (21.57) | 138 (18.04) |
| Pulmonary disease | 63 (28.51) | 269 (24.34) | 61 (29.19) | 227 (21.72) | 13 (27.66) | 44 (18.72) | 27 (17.65) | 146 (19.08) |
| Liver disease | 46 (20.81) | 228 (20.63) | 49 (23.44) | 188 (17.99) | 15 (31.91) | 45 (19.15) | 25 (16.34) | 133 (17.39) |
| Osteoporosis^c^ | 47 (21.27) | 217 (19.64) | 40 (19.14) | 163 (15.60) | 6 (12.77) | 33 (14.04) | 17 (11.11) | 104 (13.59) |
| Dyslipidemia | 83 (37.56) | 471 (42.62) | 99 (47.37) | 412 (39.43) | 16 (34.04) | 109 (46.38) | 40 (26.14) | 235 (30.72) |
| Hypertension | 138 (62.44) | 628 (56.83) | 136 (65.07) | 537 (51.39) | 26 (55.32) | 133 (56.60) | 76 (49.67) | 315 (41.18) |
| Cataract | 37 (16.74) | 214 (19.37) | 0 (0.00) | 0 (0.00) | 9 (19.15) | 33 (14.04) | 26 (16.99) | 126 (16.47) |
| Glaucoma | 20 (9.05) | 133 (12.04) | 17 (8.13) | 72 (6.89) | 0 (0.00) | 0 (0.00) | 14 (9.15) | 102 (13.33) |
| Infection | 47 (21.27) | 210 (19.00) | 23 (11.00) | 193 (18.47) | 12 (25.53) | 40 (17.02) | 30 (19.61) | 116 (15.16) |
| Myocardial infarction | 3 (1.36) | 19 (1.72) | 9 (4.31) | 24 (2.30) | 0 (0.00) | 3 (1.28) | 0 (0.00) | 0 (0.00) |
| Angina pectoris | 37 (16.74) | 198 (17.92) | 37 (17.70) | 164 (15.69) | 6 (12.77) | 39 (16.60) | 0 (0.00) | 0 (0.00) |
| Stroke | 34 (15.38) | 112 (10.14) | 26 (12.44) | 85 (8.13) | 3 (6.38) | 30 (12.77) | 0 (0.00) | 0 (0.00) |
| Cerebral hemorrhage | 4 (1.81) | 19 (1.72) | 1 (0.48) | 20 (1.91) | 1 (2.13) | 5 (2.13) | 4 (2.61) | 6 (0.78) |
| Peripheral arterial disease | 4 (1.81) | 29 (2.62) | 7 (3.35) | 15 (1.44) | 0 (0.00) | 6 (2.55) | 0 (0.00) | 0 (0.00) |
| Venous thromboembolism | 5 (2.26) | 31 (2.81) | 2 (0.96) | 22 (2.11) | 1 (2.13) | 5 (2.13) | 0 (0.00) | 0 (0.00) |
| Peptic ulcer^d^ | 0 (0.00) | 0 (0.00) | 2 (0.96) | 18 (1.72) | 2 (4.26) | 3 (1.28) | 3 (1.96) | 10 (1.31) |
| Depression | 16 (7.24) | 69 (6.24) | 10 (4.78) | 73 (6.99) | 2 (4.26) | 20 (8.51) | 5 (3.27) | 32 (4.18) |
| Insomnia | 59 (26.70) | 272 (24.62) | 60 (28.71) | 251 (24.02) | 9 (19.15) | 63 (26.81) | 36 (23.53) | 126 (16.47) |

IQR, interquartile range

^a^Matching variable.

^b^Duplicate count.
^c^Osteoporosis refers to osteoporosis with compression fracture and/or femur head necrosis.

^d^Peptic ulcer refers to peptic ulcer with gastrointestinal bleeding.


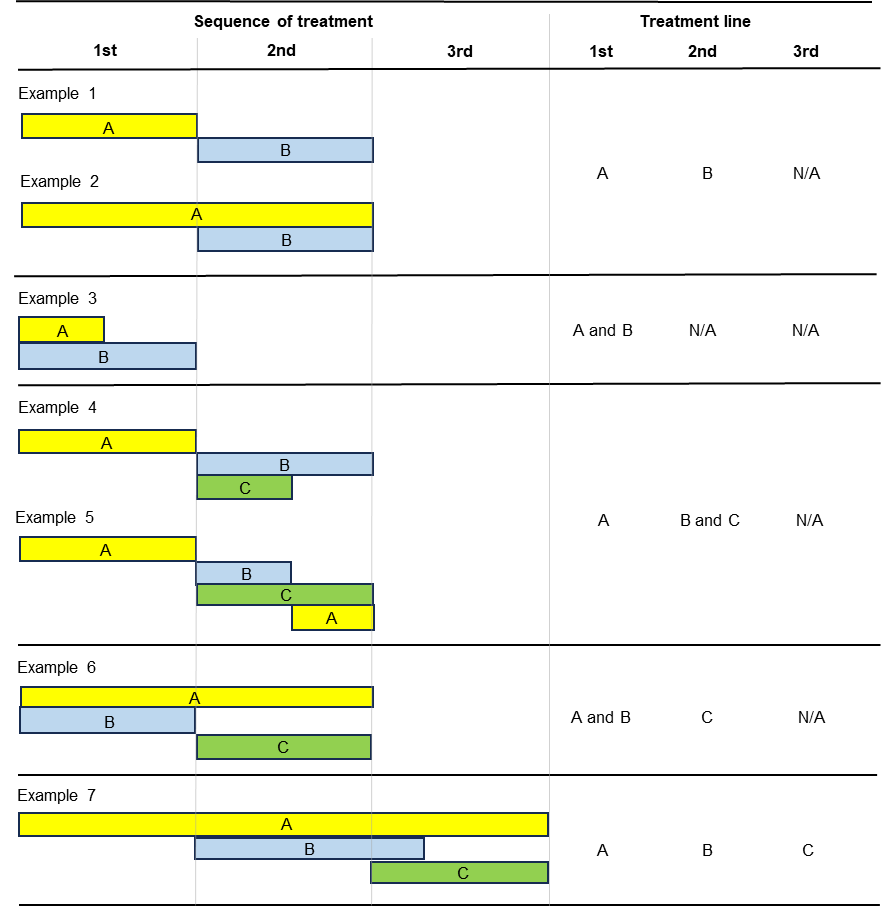


Supplementary Figure 1. Definition of treatment line.

Example9：削除

Example8：削除

A, B and C indicate examples of each different treatment.

N/A, Not Applicable.


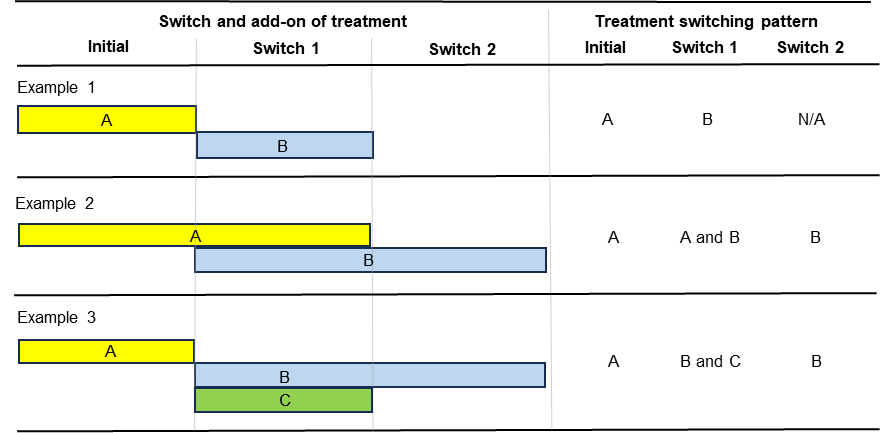


Supplementary Figure 2. Definition of treatment switching pattern.

A, B and C indicate examples of each different treatment.

N/A, Not Applicable.
